# Supplementary material for: Baseline levels and longitudinal changes in plasma Aβ42/40 among Black and white individuals
Source: Nat Commun. 2024 Jul 2;15:5539. doi: 10.1038/s41467-024-49859-w (PMC11219932; doi:10.1038/s41467-024-49859-w)

Supplemental Information
"Baseline levels and longitudinal changes in plasma Aβ42/40 among Black and white individuals" by Xiong et al.

Supplementary Table 1. Adjusted mean baseline plasma Aβ levels stratified by biomarker status as defined by CSF Aβ42/40 and amyloid PET. The linear models included the main effects of race and biomarker status and their interaction, as well as the covariates of age, sex, *APOE* ε4 carrier status, years of education, cognitive status (unimpaired, CDR 0; or impaired, CDR>0), fasting status (fasting or non-fasting), BMI, and status for hypertension and diabetes (positive or negative). The significance of the interaction of race with CSF Aβ42/40 or amyloid PET status from two-sided t-test is shown.

	Group	N Black/ white	Black adjusted mean (SE)	white adjusted mean (SE)	Difference (Black-white)	p=
**Plasma Aβ42/40**						
CSF Aβ42/40	Negative	61/440	0.1174 (0.0028)	0.1141 (0.0028)	0.0033 (0.0010)	0.61
	Positive	19/366	0.1087 (0.0033)	0.1044 (0.0029)	0.0043 (0.0017)	
Amyloid PET	Negative	71/403	0.1193 (0.0035)	0.1163 (0.0035)	0.0030 (0.0011)	0.22
	Positive	18/223	0.1139 (0.0040)	0.1081 (0.0036)	0.0057 (0.0020)	
**Plasma Aβ42**						
CSF Aβ42/40	Negative	61/440	29.65 (2.60)	30.03 (2.58)	-0.37 (0.92)	0.19
	Positive	19/366	27.85 (3.04)	25.84 (2.68)	2.00 (1.58)	
Amyloid PET	Negative	71/403	25.94 (2.92)	25.78 (2.87)	0.16 (0.88)	0.81
	Positive	18/223	21.75 (3.34)	22.03 (2.97)	-0.28 (2.04)	
**Plasma Aβ40**						
CSF Aβ42/40	Negative	61/440	226.2 (25.3)	242.5 (25.1)	-16.3 (9.0)	0.27
	Positive	19/366	246.3 (29.6)	243.3 (26.1)	3.0 (15.3)	
Amyloid PET	Negative	71/403	186.6 (28.8)	197.5 (28.3)	-10.9 (8.6)	0.37
	Positive	18/223	168.6 (33.0)	195.5 (29.3)	-26.9 (16.1)	

Supplementary Table 2. Interaction of race with covariates on plasma Aβ42/40 values. For estimated plasma Aβ42/40 values, the interaction of race with each covariate is shown. The linear models additionally included the main effects of race and the covariates of age, sex, *APOE ε*4 carrier status, years of education, cognitive status (unimpaired, CDR 0; or impaired, CDR>0), fasting status (fasting or non-fasting), BMI, and status for hypertension and diabetes (positive or negative). The significance of interaction of race with the covariate from two-sided t-test is shown.

Interacting covariate	Group	N Black/ white	Black adjusted mean (SE)	white adjusted mean (SE)	Difference (Black-white)	p=
Age	≤70.62 years	124/567	0.1028 (0.0019)	0.0982 (0.0017)	0.0046	0.92
	>70.62 years	90/546	0.0995 (0.0020)	0.0950 (0.0017)	0.0045	
Sex	Male	60/531	0.1176 (0.0032)	0.1129 (0.0031)	0.0048	0.87
	Female	154/582	0.1200 (0.0031)	0.1155 (0.0030)	0.0045	
APOE ε4	Non-carrier	111/638	0.1199 (0.0030)	0.1156 (0.0030)	0.0043	0.67
	Carrier	103/475	0.1149 (0.0030)	0.1100 (0.0029)	0.0049	
Years of education	≤12 years	50/178	0.1227 (0.0028)	0.1182 (0.0026)	0.0046	0.98
	>12 years	164/935	0.1231 (0.0026)	0.1185 (0.0025)	0.0045	
Cognitive status	Unimpaired	51/333	0.1248 (0.0029)	0.1192 (0.0029)	0.0056	0.017
	Impaired	163/780	0.1176 (0.0032)	0.1159 (0.0030)	0.0017	
BMI	≤30	33/372	0.1204 (0.0033)	0.1155 (0.0030)	0.0049	0.82
	>30	181/741	0.1203 (0.0029)	0.1158 (0.0029)	0.0045	
Hypertension	Negative	74/634	0.1201 (0.0031)	0.1156 (0.0030)	0.0045	0.94
	Positive	140/479	0.1206 (0.0031)	0.1160 (0.0031)	0.0046	
Diabetes	Negative	159/102	0.1206 (0.0030)	0.1156 (0.0030)	0.0050	0.23
	Positive	55/91	0.1183 (0.0032)	0.1154 (0.0032)	0.0028	

Supplementary Table 3. Interaction of race with covariates on plasma Aβ42. For estimated plasma Aβ42 values, the interaction of race with each covariate is shown. The linear models additionally included the main effects of race and the covariates of age, sex, *APOE ε*4 carrier status, years of education, cognitive status (unimpaired, CDR 0; or impaired, CDR>0), fasting status (fasting or non-fasting), BMI, and status for hypertension and diabetes (positive or negative). The significance of interaction of race with the covariate from two-sided t-test is shown.

**Interacting covariate**	**Group**	**N Black/ white**	**Black adjusted mean (SE)**	**white adjusted mean (SE)**	**Difference (Black-white)**	**p=**
Age	≤70.62 years	124/567	39.58 (1.50)	40.78 (1.37)	-1.20 (0.78)	0.031
	>70.62 years	90/546	44.01 (1.61)	42.70 (1.35)	1.32 (0.92)	
Sex	Male	60/531	24.25 (2.57)	24.21 (2.47)	0.03 (1.04)	0.82
	Female	154/582	26.15 (2.45)	26.39 (2.37)	-0.25 (0.73)	
APOE ε4	Non-carrier	111/638	26.30 (2.43)	26.38 (2.37)	-0.08 (0.81)	0.89
	Carrier	103/475	23.49 (2.43)	23.73 (2.33)	-0.24 (0.85)	
Years of education	≤12 years	50/178	27.20 (2.23)	27.83 (2.08)	-0.63 (1.22)	0.66
	>12 years	164/935	27.98 (2.09)	28.01 (2.00)	-0.03 (0.68)	
Cognitive status	Unimpaired	51/333	27.77 (2.32)	27.81 (2.28)	-0.05 (0.69)	0.74
	Impaired	163/780	25.97 (2.55)	26.45 (2.37)	-0.48 (1.15)	
BMI	≤30	33/372	28.34 (2.64)	26.15 (2.37)	2.19 (1.38)	0.058
	>30	181/741	27.06 (2.34)	27.73 (2.30)	-0.67 (0.66)	
Hypertension	Negative	74/634	26.28 (2.50)	26.41 (2.37)	-0.13 (0.94)	0.97
	Positive	140/479	28.18 (2.49)	28.35 (2.45)	-0.17 (0.77)	
Diabetes	Negative	159/102	26.02 (2.42)	26.40 (2.37)	-0.38 (0.67)	0.44
	Positive	55/91	28.97 (2.57)	28.22 (2.52)	0.75 (1.30)	

Supplementary Table 4. Interaction of race with covariates on plasma Aβ40. For estimated plasma Aβ40 values, the interaction of race with each covariate is shown. The linear mixed effects models additionally included the main effects of race and the covariates of age, sex, *APOE ε*4 carrier status, years of education, cognitive status (unimpaired, CDR 0; or impaired, CDR>0), fasting status (fasting or non-fasting), BMI, and status for hypertension and diabetes (positive or negative). The significance of interaction of race with the covariate from two-sided t-test is shown.

Interacting covariate	Group	N Black/ white	Black adjusted mean (SE)	white adjusted mean (SE)	Difference (Black-white)	p=
Age	≤70.62 years	124/567	387.3 (14.5)	419.0 (13.2)	-31.8 (7.5)	0.027
	>70.62 years	90/546	445.7 (15.5)	452.7 (13.0)	-7.0 (8.9)	
Sex	Male	60/531	170.7 (24.3)	190.4 (23.3)	-19.7 (9.8)	0.81
	Female	154/582	176.8 (23.2)	199.3 (22.4)	-22.6 (6.9)	
APOE ε4	Non-carrier	111/638	179.5 (23.0)	198.8 (22.4)	-19.3 (7.6)	0.64
	Carrier	103/475	172.2 (23.0)	196.5 (22.1)	-24.3 (8.1)	
Years of education	≤12 years	50/178	175.7 (21.1)	200.7 (19.6)	-25.0 (11.5)	0.73
	>12 years	164/935	180.4 (19.8)	200.9 (18.9)	-20.5 (6.5)	
Cognitive status	Unimpaired	51/333	171.4 (22.0)	197.0 (21.6)	-25.6 (6.5)	0.20
	Impaired	163/780	188.2 (24.1)	197.9 (22.4)	-9.7 (10.9)	
BMI	≤30	33/372	196.4 (25.0)	197.2 (22.4)	-0.8 (13.0)	0.076
	>30	181/741	184.8 (22.2)	211.0 (21.8)	-26.2 (6.3)	
Hypertension	Negative	74/634	181.3 (23.6)	199.3 (22.4)	-18.0 (8.9)	0.59
	Positive	140/479	193.3 (23.5)	217.3 (23.2)	-24.0 (7.2)	
Diabetes	Negative	159/1022	174.2 (22.9)	199. 4 (22.4)	-25.2 (6.3)	0.18
	Positive	55/91	212.2 (24.3)	219.2 (23.8)	-7.0 (12.3)	

Supplementary Table 5. Estimated annual rates of longitudinal change in plasma Aβ for groups of Black and white individuals as a function of both race and biomarker status as defined by CSF Aβ42/40 and amyloid PET. The linear mixed effects models included the main effects of race, biomarker status, time, and their interactions, as well as the covariates of age, sex, *APOE ε*4 carrier status, years of education, cognitive status (unimpaired, CDR 0; or impaired, CDR>0), fasting status (fasting or non-fasting), BMI, and status for hypertension and diabetes (positive or negative). The significance of the interaction of race with CSF Aβ42/40 or amyloid PET status on the slope (i.e., annual rate of change) from two-sided t-test is shown.

	Group	N Black/ white	Black slope (SE)	white slope (SE)	Difference (Black-white)	p=
**Plasma Aβ42/40**						
CSF Aβ42/40	Negative	39/288	-0.00035 (0.00018)	-0.00077 (0.00007)	0.00041 (0.00018)	0.56
	Positive	9/165	-0.00050 (0.00057)	-0.00056 (0.00011)	0.00006 (0.00058)	
Amyloid PET	Negative	43/256	-0.00039 (0.00021)	-0.00083 (0.00008)	0.00044 (0.00022)	0.28
	Positive	8/93	-0.00078 (0.00057)	-0.00054 (0.00014)	-0.00024 (0.00059)	
**Plasma Aβ42**						
CSF Aβ42/40	Negative	39/288	0.4369 (0.1508)	0.1276 (0.0630)	0.3094 (0.1568)	0.76
	Positive	9/165	0.3005 (0.4798)	0.1448 (0.0913)	0.1557 (0.4873)	
Amyloid PET	Negative	43/256	0.3906 (0.1713)	0.1219 (0.0670)	0.2687 (0.1777)	0.64
	Positive	8/93	0.7354 (0.4735)	0.2274 (0.1178)	0.5080 (0.4848)	
**Plasma Aβ40**						
CSF Aβ42/40	Negative	39/288	4.838 (1.382)	4.487 (0.575)	0.351 (1.438)	0.99
	Positive	9/165	4.875 (4.346)	4.444 (0.830)	0.431 (4.414)	
Amyloid PET	Negative	43/256	4.607 (1.598)	4.745 (0.624)	-0.138 (1.662)	0.18
	Positive	8/93	11.244 (4.349)	4.990 (1.094)	6.254 (4.455)	

Supplementary Table 6. Estimated annual rates of longitudinal change in plasma Aβ42/40 for groups of Black and white individuals as a function of main covariates. For estimated rates of change, the interaction of race with each covariate is shown. The models included the main effects of race, covariate, time, and their interactions, as well as remaining covariates from age, sex, *APOE ε*4 carrier status, years of education, cognitive status (unimpaired, CDR 0; or impaired, CDR>0), fasting status (fasting or non-fasting), BMI, and status for hypertension and diabetes (positive or negative). The significance of interaction of race with the covariate on the slope from two-sided t-test is shown.

Interacting covariate	Group	N Black/ white	Black slope (SE)	white slope (SE)	Difference (Black-white)	p=
Age	≤70.62 years	70/334	-0.00048 (0.00017)	-0.00068 (0.00008)	0.00020 (0.00018)	0.41
	>70.62 years	42/232	-0.00066 (0.00028)	-0.00058 (0.00011)	-0.00008 (0.00029)	
Sex	Male	35/270	-0.00036 (0.00023)	-0.00065 (0.00009)	0.00030 (0.00024)	0.32
	Female	77/296	-0.00068 (0.00019)	-0.00067 (0.00009)	-0.00001 (0.00020)	
APOE ε4	Non-carrier	61/347	-0.00042 (0.00018)	-0.00072 (0.00008)	0.00030 (0.00019)	0.095
	Carrier	51/219	-0.00075 (0.00024)	-0.00053 (0.00010)	-0.00021 (0.00025)	
Years of education	≤12 years	21/81	-0.00006 (0.00035)	-0.00060 (0.00015)	0.00055 (0.00038)	0.21
	>12 years	91/485	-0.00063 (0.00016)	-0.00067 (0.00007)	0.00004 (0.00016)	
Cognitive status	Unimpaired	97/478	-0.00058 (0.00016)	-0.00065 (0.00007)	0.00007 (0.00016)	0.47
	Impaired	15/88	-0.00026 (0.00044)	-0.00069 (0.00018)	0.00043 (0.00047)	
BMI	≤30	13/175	-0.00099 (0.00042)	-0.00070 (0.00011)	-0.00029 (0.00043)	0.34
	>30	99/391	-0.00048 (0.00016)	-0.00063 (0.00008)	0.00015 (0.00016)	
Hypertension	Negative	39/334	-0.00048 (0.00023)	-0.00066 (0.00008)	0.00018 (0.00023)	0.73
	Positive	73/232	-0.00059 (0.00019)	-0.00066 (0.00010)	0.00007 (0.00020)	
Diabetes	Negative	89/523	-0.00049 (0.00016)	-0.00066 (0.00007)	0.00017 (0.00016)	0.41
	Positive	23/43	-0.00084 (0.00034)	-0.00067 (0.00020)	-0.00018 (0.00039)	

Supplementary Table 7. Estimated annual rates of longitudinal change in plasma Aβ42 for groups of Black and white individuals as a function of main covariates. For estimated rates of change, the interaction of race with each covariate is shown. The models included the main effects of race, covariate, time, and their interactions, as well as remaining covariates from age, sex, *APOE ε*4 carrier status, years of education, cognitive status (unimpaired, CDR 0; or impaired, CDR>0), fasting status (fasting or non-fasting), BMI, and status for hypertension and diabetes (positive or negative). The significance of interaction of race with the covariate on the slope is shown.

Interacting covariate	Group	Black slope (SE)	white slope (SE)	Difference (Black-white)	p=
Age	≤70.62 years	0.5155 (0.1360)	0.0351 (0.0622)	0.4804 (0.1408)	0.0059
	>70.62 years	0.2218 (0.2268)	0.4902 (0.0896)	-0.2683 (0.2333)	
Sex	Male	0.6340 (0.1864)	0.1790 (0.0752)	0.4550 (0.1945)	0.20
	Female	0.2778 (0.1549)	0.1458 (0.0706)	0.1320 (0.1593)	
APOE ε4	Non-carrier	0.3872 (0.1506)	0.1372 (0.0664)	0.2499 (0.1547)	0.96
	Carrier	0.4771 (0.1920)	0.2153 (0.0833)	0.2618 (0.2015)	
Years of education	≤12 years	0.7109 (0.2881)	0.3275 (0.1215)	0.3834 (0.3080)	0.65
	>12 years	0.3680 (0.1324)	0.1363 (0.0601)	0.2318 (0.1339)	
Cognitive status	Unimpaired	0.4176 (0.1279)	0.1611 (0.0589)	0.2565 (0.1298)	0.98
	Impaired	0.4550 (0.3585)	0.2113 (0.1460)	0.2437 (0.380)	
BMI	≤30	0.6183 (0.3435)	0.0947 (0.0862)	0.5236 (0.3526)	0.39
	>30	0.3965 (0.1302)	0.1961 (0.0658)	0.2004 (0.1330)	
Hypertension	Negative	0.3138 (0.1827)	0.0662 (0.0672)	0.2477 (0.1874)	0.77
	Positive	0.4932 (0.1540)	0.3187 (0.0792)	0.1744 (0.1631)	
Diabetes	Negative	0.4301 (0.1325)	0.1471 (0.0592)	0.2830 (0.1339)	0.47
	Positive	0.3830 (0.2809)	0.3498 (0.1580)	0.0332 (0.3171)	

Supplementary Table 8. Estimated annual rates of longitudinal change in plasma Aβ40 for groups of Black and white individuals as a function of main covariates. For estimated rates of change, the interaction of race with each covariate is shown. The models included the main effects of race, covariate, time, and their interactions, as well as remaining covariates from age, sex, *APOE ε*4 carrier status, years of education, cognitive status (unimpaired, CDR 0; or impaired, CDR>0), fasting status (fasting or non-fasting), BMI, and status for hypertension and diabetes (positive or negative). The significance of interaction of race with the covariate on the slope is shown.

Interacting covariate	Group	Black slope (SE)	white slope (SE)	Difference (Black-white)	p=
Age	≤70.62 years	6.641 (1.248)	3.344 (0.570)	3.297 (1.294)	0.018
	>70.62 years	5.317 (2.068)	7.874 (0.818)	-2.557 (2.129)	
Sex	Male	7.195 (1.714)	4.989 (0.689)	2.206 (1.789)	0.60
	Female	5.281 (1.416)	4.282 (0.647)	0.999 (1.458)	
APOE ε4	Non-carrier	5.002(1.383)	4.556 (0.608)	0.447 (1.421)	0.27
	Carrier	7.781 (1.754)	4.760 (0.764)	3.021 (1.843)	
Years of education	≤12 years	6.547 (2.646)	6.162 (1.117)	0.385 (2.830)	0.69
	>12 years	5.969 (1.215)	4.371 (0.551)	1.598 (1.231)	
Cognitive status	Unimpaired	6.051 (1.172)	4.486 (0.539)	1.565 (1.191)	0.68
	Impaired	6.253 (3.277)	6.189 (1.333)	0.064 (3.473)	
BMI	≤30	9.526 (3.140)	3.915 (0.789)	5.611 (3.223)	0.15
	>30	5.587 (1.192)	4.920 (0.602)	0.666 (1.219)	
Hypertension	Negative	4.150 (1.662)	3.478	0.671 (1.705)	0.92
	Positive	7.297 (1.399)	6.411 (0.720)	0.886 (1.483)	
Diabetes	Negative	5.860 (1.211)	4.470 (0.541)	1.390 (1.225)	0.84
	Positive	7.031 (2.573)	6.284 (1.448)	0.747 (2.906)	

Supplementary Figure 1. Partial correlations of plasma Aβ biomarkers with CSF biomarkers, amyloid PET, and cognitive composites. Partial Spearman correlations between plasma Aβ biomarkers and CSF biomarkers, amyloid PET, or cognitive composites are shown for Black (**A**) and white (**B**) individuals. Partial Spearman correlations adjusted for the effects of age, sex, *APOE* ε4 carrier status, years of education, cognitive status (unimpaired, CDR 0; or impaired, CDR>0), fasting status if plasma biomarker (fasting or non-fasting), BMI, and status for hypertension and diabetes (positive or negative). Racial differences in partial correlations (Black-white) are shown in (**C).** Only significant partial correlations or differences (raw p<0.05 from two-sided normal test) are shown, and no racial differences were significant after FDR-adjustment at 5%. Colored blocks visualize correlation direction and magnitude, with warm to cold colors representing most negative correlations to most positive correlations and darker color and larger block size corresponding to greater absolute magnitudes. Source data are provided as a Source Data file.

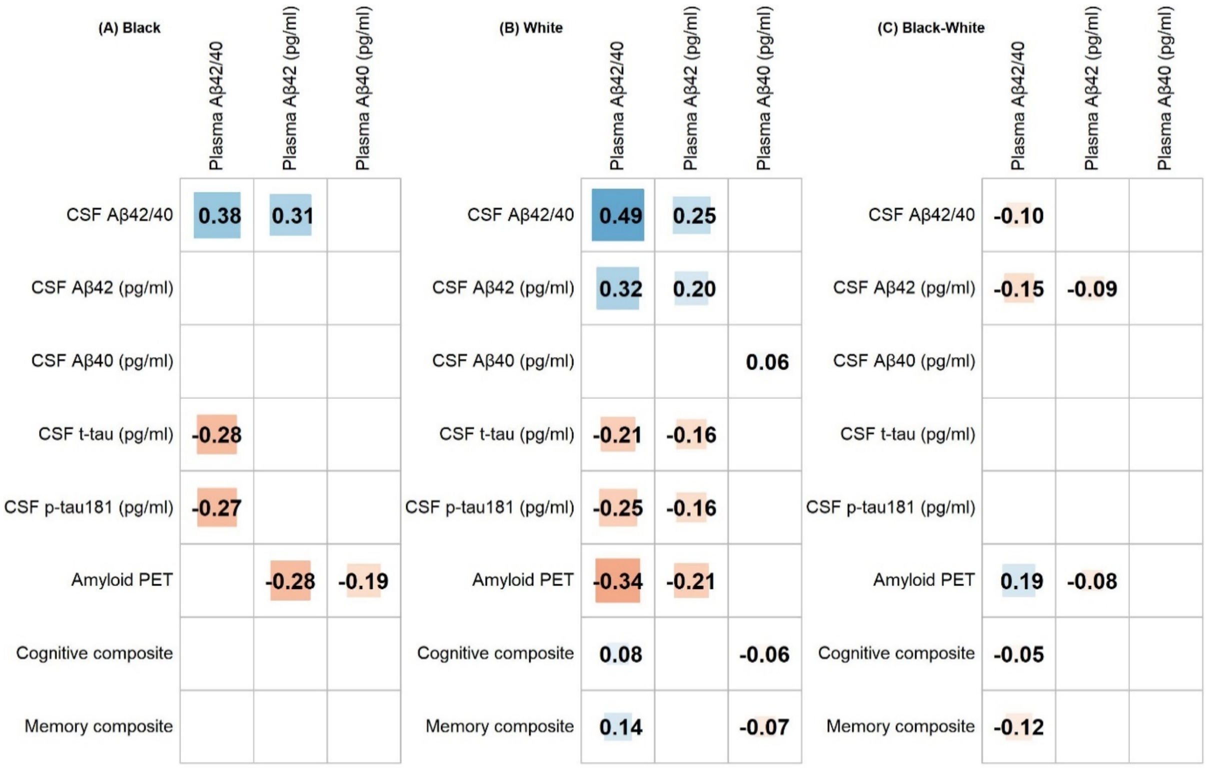

Supplementary Figure 2. Fitted longitudinal trajectories of plasma markers. The fitted longitudinal trajectories of plasma Aβ42/40 (A), Aβ42 (B), and Aβ40 (C) as a function of race and cognitive status at baseline, as predicted from linear mixed effects model. Colors represent the following: grey, white participants; red, Black participants; solid line, cognitively normal; dashed line; cognitively impaired. Source data are provided as a Source Data file.

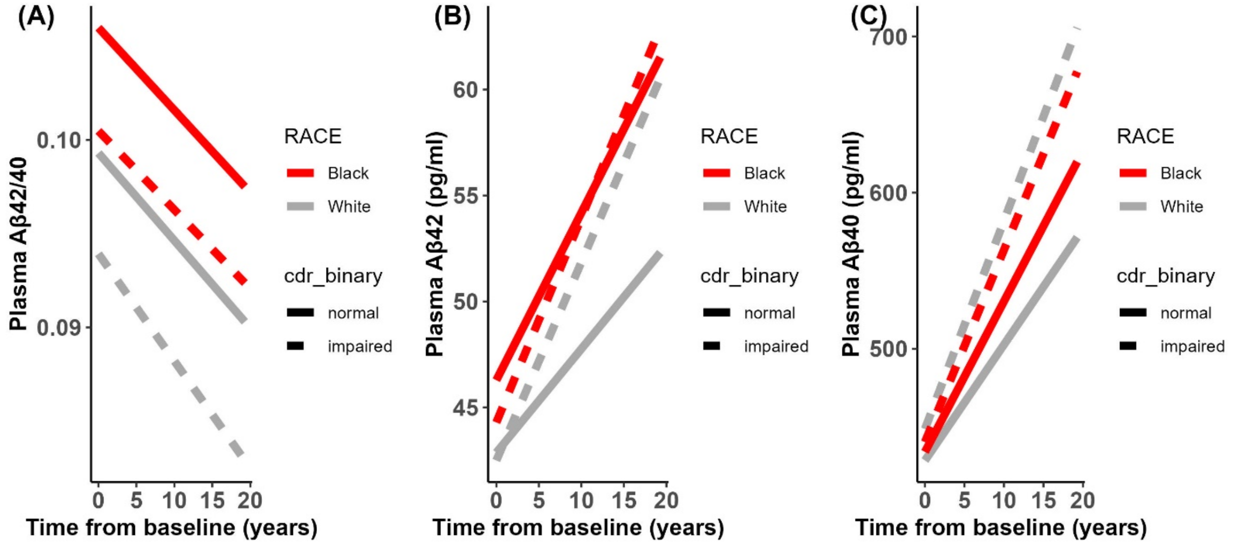

Supplement: Supplementary file 1 — Supplementary Information [file 41467_2024_49859_MOESM1_ESM.pdf]
