# Supplementary material for: Baseline levels and longitudinal changes in plasma Aβ42/40 among Black and white individuals
Source: Nat Commun. 2024 Jul 2;15:5539. doi: 10.1038/s41467-024-49859-w (PMC11219932; doi:10.1038/s41467-024-49859-w)
Supplement: Supplementary file 3 — Reporting Summary [file 41467_2024_49859_MOESM3_ESM.pdf]

nature portfolio

Corresponding author(s): Suzanne Schindler
Last updated by author(s): 5/16/2024

Reporting Summary

Nature Portfolio wishes to improve the reproducibility of the work that we publish. This form provides structure for consistency and transparency in reporting. For further information on Nature Portfolio policies, see our Editorial Policies and the Editorial Policy Checklist.

Statistics

For all statistical analyses, confirm that the following items are present in the figure legend, table legend, main text, or Methods section.

n/a	Confirmed	
☐	☒	The exact sample size (n) for each experimental group/condition, given as a discrete number and unit of measurement
☐	☒	A statement on whether measurements were taken from distinct samples or whether the same sample was measured repeatedly
☐	☒	The statistical test(s) used AND whether they are one- or two-sided<br>*Only common tests should be described solely by name; describe more complex techniques in the Methods section.*
☐	☒	A description of all covariates tested
☐	☒	A description of any assumptions or corrections, such as tests of normality and adjustment for multiple comparisons
☐	☒	A full description of the statistical parameters including central tendency (e.g. means) or other basic estimates (e.g. regression coefficient) AND variation (e.g. standard deviation) or associated estimates of uncertainty (e.g. confidence intervals)
☐	☒	For null hypothesis testing, the test statistic (e.g. F, t, r) with confidence intervals, effect sizes, degrees of freedom and P value noted<br>*Give P values as exact values whenever suitable.*
☒	☐	For Bayesian analysis, information on the choice of priors and Markov chain Monte Carlo settings
☐	☒	For hierarchical and complex designs, identification of the appropriate level for tests and full reporting of outcomes
☐	☒	Estimates of effect sizes (e.g. Cohen's d, Pearson's r), indicating how they were calculated

Our web collection on statistics for biologists contains articles on many of the points above.

Software and code

Policy information about availability of computer code

Data collection	We used publicly available software, Rstudio, for the analyses. All software used in this study is described in the Methods section and the accompanying Reporting Summary
Data analysis	We used publicly available software, Rstudio, version 2023.9.1.494 running R version 4.2.1, for the analyses. All software used in this study is described in the Methods section and the accompanying Reporting Summary

For manuscripts utilizing custom algorithms or software that are central to the research but not yet described in published literature, software must be made available to editors and reviewers. We strongly encourage code deposition in a community repository (e.g. GitHub). See the Nature Portfolio guidelines for submitting code & software for further information.

Data

Policy information about availability of data

All manuscripts must include a data availability statement. This statement should provide the following information, where applicable:

- Accession codes, unique identifiers, or web links for publicly available datasets
- A description of any restrictions on data availability
- For clinical datasets or third party data, please ensure that the statement adheres to our policy

Anonymized data that support the findings of this study are available from the corresponding author and the first author, upon request from any qualified investigator.

Research involving human participants, their data, or biological material

Policy information about studies with human participants or human data. See also policy information about sex, gender (identity/presentation), and sexual orientation and race, ethnicity and racism.

Reporting on sex and gender	sex was analyzed in all major analyses
Reporting on race, ethnicity, or other socially relevant groupings	race was the major analysis of the study
Population characteristics	Table 1 describe all the study population characteristics
Recruitment	Section 2.1 described the recruitment methods by all centers participating in this study
Ethics oversight	The Washington University Human Research Protection Office approved the current study with additional approvals from the Institutional Review Boards of the other sites.

Note that full information on the approval of the study protocol must also be provided in the manuscript.

Field-specific reporting

Please select the one below that is the best fit for your research. If you are not sure, read the appropriate sections before making your selection.

☒ Life sciences ☐ Behavioural & social sciences ☐ Ecological, evolutionary & environmental sciences

For a reference copy of the document with all sections, see nature.com/documents/nr-reporting-summary-flat.pdf

Life sciences study design

All studies must disclose on these points even when the disclosure is negative.

Sample size	The research-based study cohort included all (a total of 324 Black and 1,547 White participants) who had any plasma Aβ measures at the time of analysis from the Study of Race to Understand Alzheimer Biomarkers (SORTOUT-AB; NIH/NIA R01 AG067505), which aims to understand potential racial differences in harmonized biomarker data collected by multiple research studies of memory and aging in middle-aged and older individuals.
Data exclusions	no data were excluded.
Replication	we reported likely the largest longitudinal C2N plasma abeta biomarker data set in Black participants by processing all samples currently available from 3 ADRCs, no replication data are currently available.
Randomization	this is an observational study to compare plasma Abeta, both cross-sectionally and longitudinally, in Black and White individuals. no randomization is needed.
Blinding	blinding is not possible because race was self-identified in this study

Reporting for specific materials, systems and methods

We require information from authors about some types of materials, experimental systems and methods used in many studies. Here, indicate whether each material, system or method listed is relevant to your study. If you are not sure if a list item applies to your research, read the appropriate section before selecting a response.

Materials & experimental systems

n/a	Involved in the study	
☒	☐	Antibodies
☒	☐	Eukaryotic cell lines
☒	☐	Palaeontology and archaeology
☒	☐	Animals and other organisms
☐	☒	Clinical data
☒	☐	Dual use research of concern
☒	☐	Plants

Methods

n/a	Involved in the study	
☒	☐	ChIP-seq
☒	☐	Flow cytometry
☒	☐	MRI-based neuroimaging

Clinical data

Policy information about clinical studies

All manuscripts should comply with the ICMJEguidelines for publication of clinical research and a completedCONSORT checklist must be included with all submissions.

Clinical trial registration	this is an observational and natural history study, not a clinical trial
Study protocol	access to study protocol can be requested to the first author
Data collection	see section 2.4. Blood and CSF collection and analysis
Outcomes	the primary outcomes are plasma Ab42, Ab40, and Ab42/40 ratio.

Plants

Seed stocks	*Report on the source of all seed stocks or other plant material used. If applicable, state the seed stock centre and catalogue number. If plant specimens were collected from the field, describe the collection location, date and sampling procedures.*
Novel plant genotypes	*Describe the methods by which all novel plant genotypes were produced. This includes those generated by transgenic approaches, gene editing, chemical/radiation-based mutagenesis and hybridization. For transgenic lines, describe the transformation method, the number of independent lines analyzed and the generation upon which experiments were performed. For gene-edited lines, describe the editor used, the endogenous sequence targeted for editing, the targeting guide RNA sequence (if applicable) and how the editor was applied.*
Authentication	*Describe any authentication procedures for each seed stock used or novel genotype generated. Describe any experiments used to assess the effect of a mutation and, where applicable, how potential secondary effects (e.g. second site T-DNA insertions, mosiacism, off-target gene editing) were examined.*
